# Interpersonal trauma: psychodynamic psychotherapy and neurobiology

**DOI:** 10.1080/20008198.2017.1351202

**Published:** 2017-09-29

**Authors:** Helen Lazaratou

**Affiliations:** ^a^ Department of Psychiatry, Medical School, National and Kapodistrian University of Athens, Eginiteio Hospital, Athens, Greece

**Keywords:** Early trauma, interpersonal trauma, psychodynamic psychotherapy, neurobiology

## Abstract

In psychodynamic theory, trauma is associated with a life event, which is defined by its intensity, by the inability of the person to respond adequately and by its pathologic long-lasting effects on the psychic organization. In this paper, we describe how neurobiological changes link to psychodynamic theory.

Initially, Freud believed that all types of neurosis were the result of former traumatic experiences, mainly in the form of sexual trauma. According to the first Freudian theory (1890–1897), hysteric patients suffer mainly from relevant memories. In his later theory of ‘differed action’, i.e., the retroactive attribution of sexual or traumatic meaning to earlier events, Freud links the consequences of sexual trauma in childhood with the onset of pathology in adulthood (Boschan, 2008).

The transmission of trauma from parents to children may take place from one generation to the other. The trauma that is being experienced by the child has an interpersonal character and is being reinforced by the parents’ own traumatic experience. The subject’s interpersonal exposure through the relationship with the direct victims has been recognized as a risk factor for the development of a post-traumatic stress disorder.

Trauma may be transmitted from the mother to the foetus during the intrauterine life (Opendak & Sullivan, 2016). Empirical studies also demonstrate that in the first year of life infants that had witnessed violence against their mothers presented symptoms of a post-traumatic disorder. Traumatic symptomatology in infants includes eating difficulties, sleep disorders, high arousal level and excessive crying, affect disorders and relational problems with adults and peers. Infants that are directly dependant to the caregiver are more vulnerable and at a greater risk to suffer interpersonal trauma and its neurobiological consequences (Opendak & Sullivan, 2016). In older children symptoms were more related to the severity of violence they had been exposed to than to the mother’s actual emotional state, which shows that the relationship between mother’s and child’s trauma is different in each age stage.

The type of attachment and the quality of the mother-child interactional relationship contribute also to the transmission of the trauma. According to Fonagy (2003), the mother who is experiencing trauma is no longer a source of security and becomes a source of danger. Thus, the mentalization ability may be destroyed by an attachment figure, which caused to the child enough stress related to its own thoughts and emotions to an extent, that the child avoids thoughts about the other’s subjective experience.

At a neurobiological level, many studies have shown that the effects of environmental stress on the brain are being mediated through molecular and cellular mechanisms. More specifically, trauma causes changes at a chemical and anatomical level resulting in transforming the subject’s response to future stress.

The imprinting mechanisms of traumatic experiences are directly related to the activation of the neurobiological circuits associated with emotion, in which amygdala play a central role. The traumatic experiences are strongly encoded in memory and difficult to be erased. Early stress may result in impaired cognitive function related to disrupted functioning of certain areas of the hippocampus in the short or long term.

Infants or young children that have suffered a traumatic experience may are unable to recollect events in a conscious way. However, they may maintain latent memory of the reactions to the experience and the intensity of the emotion. The neurobiological data support the ‘deferred action’ of the psychodynamic theory according which when the impact of early interpersonal trauma is so pervasive, the effects can transcend into later stages, even after the trauma has stopped.

The two approaches, psychodynamic and neurobiological, are not opposite, but complementary. Psychodynamic psychotherapists and neurobiologists, based on extended theoretical bases, combine data and enrich the understanding of psychiatric disorders in childhood. The study of interpersonal trauma offers a good example of how different approaches, biological and psychodynamic, may come closer and possibly be unified into a single model, which could result in more effective therapeutic approaches.
